# Variation in susceptibility of different breeds of sheep to *Mycobacterium avium* subspecies *paratuberculosis* following experimental inoculation

**DOI:** 10.1186/s13567-017-0440-7

**Published:** 2017-06-17

**Authors:** D. J. Begg, A. C. Purdie, K. de Silva, N. K. Dhand, K. M. Plain, R. J. Whittington

**Affiliations:** Farm Animal and Veterinary Public Health, Sydney School of Veterinary Science and School of Life and Environmental Sciences, Faculty of Science, The University of Sydney, 425 Werombi Rd, Camden, NSW 2570 Australia

## Abstract

Exposure to *Mycobacterium avium* subspecies *paratuberculosis* (MAP) does not always lead to Johne’s disease. Understanding differences in disease susceptibility of individual animals is a key aspect to controlling mycobacterial diseases. This study was designed to examine the susceptibility or resistance of various breeds of sheep to MAP infection. Merino, Suffolk first cross Merino, Border Leicester, and Poll Dorset sheep were orally inoculated with MAP and monitored for 14 months. Clinical disease occurred more frequently in the Merino (42%) and Suffolk first cross Merino (36%) compared to the Border Leicester (12%) and Poll Dorset (11%) breeds. Infection risk, as determined by culture of gut and associated lymphoid tissues, ranged from 75% for the Suffolk first cross Merino to 47% for the Poll Dorset sheep. Significant differences were identified in the site in the intestines of the most severe histopathological lesions and the immune responses to infection between the breeds. However, there was no difference in faecal MAP shedding by clinical cases between breeds. All breeds tested were susceptible to MAP infection, as determined by infection and clinical disease development, although there were differences in the proportions of diseased animals between the breeds. Poll Dorset and Border Leicester sheep were more resilient to MAP infection but there was evidence that more animals could have developed disease if given more time. These findings provide evidence of potential differential disease susceptibility between breeds, further our understanding of disease pathogenesis and risks of disease spread, and may have an influence on control programs for *paratuberculosis*.

## Introduction

*Mycobacterium avium* subspecies *paratuberculosis* (MAP) causes Johne’s disease in ruminant hosts globally and is a source of economic loss. For this reason, and because of a potential zoonotic link [1] control programs for MAP have been implemented in many developed countries. These are based on hygiene measures and the removal of cases from affected herds and flocks. Increasing the level of resistance at population level through vaccination is also possible [2, 3]. However, increasing the level of genetic resistance or resilience to MAP infection is relatively unstudied. Rather than being a slowly progressive and eventually fatal infection in all cases, it is now known that not all animals exposed to MAP develop Johne’s disease (JD) and some appear to clear the infection spontaneously [4]. Furthermore, it is suspected that some breeds of ruminants are more resistant to MAP infection than others, but objective information is limited as no controlled experimental infection trials have been conducted to directly compare different breeds. Some breeds may be more susceptible than others, based on anecdotal evidence and one cross sectional survey of farmers, which suggested that fine wool Merino sheep were more likely to develop clinical JD [5]. It is known that many different breeds of sheep can develop clinical JD including Merino, Churra, Blackface, Texel, Bleu du Maine, East Friesian, Romney, Highland cross and Rocky Mountain bighorn sheep [6–9].

Other studies have shown that genetics may play a role in the susceptibility to JD in ruminants [10, 11] and there is evidence that different breeds can vary in the amount of serum antibody produced in response to MAP exposure [12, 13]. It is difficult to determine in these studies whether the differences were due to differential antibody production, different stages of disease, and differences in the response to infection or were due to different trading patterns, which could result in differing levels of MAP exposure.

The aim of this study was to examine the susceptibility to MAP infection of four breeds of sheep. Relevant sheep breeds were identified following consultation with industry experts in Australia. The representatives of the Sheepmeat Council of Australia and WoolProducers Australia provided advice on which breeds to examine, those most commonly used in Australia. Lambs of each breed from MAP-free flocks were inoculated and the animals were monitored for disease development with the severity of disease confirmed. The findings indicate that all of the breeds tested can develop JD, but differences in susceptibility may exist between breeds.

## Materials and methods

### Animals

The use of animals in this study was approved by the University of Sydney Animal Ethics committee protocol number N00/10-2010/3/5372.

One hundred and sixty-nine sheep comprising more than 40 sheep of each of four different breeds were purchased from farms participating in the Australian Market Assurance Program for *paratuberculosis*. All farms were in the Armidale region of New South Wales, Australia, and had a program score of Monitored Negative 3. This is the highest assurance that a farm is free of MAP infected sheep [14]. The farms were also chosen as they had a similar lambing time so that all lambs were approximately 5 months of age at inoculation.

Forty-six Merino, 41 Poll Dorset, 41 Border Leicester and 41 Suffolk first cross Merino lambs were used in the experiment. The Merino breed was used as a control group, as previous experimental inoculation trials have been run in this breed with the same methodology [15]. The old British breeds of Poll Dorset, Border Leicester and Suffolk were chosen as they are the most commonly farmed sheep in Australia after the Merino. Due to an unexpected operational issue, pure breed Suffolk lambs could not be supplied, and only Suffolk first cross Merino lambs were available. All suppliers were asked to provide castrated males, but when the sheep arrived it was discovered that the Poll Dorset and Border Leicester lambs were predominantly females.

The animals for each breed were allocated by systematic sampling into two groups by drafting off every fourth Merino or every tenth animal of the other breeds. The first group consisted of 10 Merino, 5 Poll Dorset, 5 Border Leicester and 5 Suffolk first cross Merino lambs. This group was used as an un-inoculated control group. The second group consisted of 36 lambs of each breed, and these were inoculated with MAP. The control animals were held on separate pasture adjacent to the inoculated animals; the pastures housing the controls had not held MAP-infected sheep in the past. The animals were managed under conventional Australian sheep farming conditions by grazing in open paddocks on unimproved pasture with reticulated water in elevated troughs; supplementary feeding with grain/lucerne chaff was provided as necessary. The lambs from different breeds were grazed together based on their status as controls or MAP-inoculated.

### Experimental inoculation of the lambs

The oral inoculations of the sheep were as described by Begg et al. [15] using a pure culture S strain of MAP (Telford 9.2). Three doses were delivered over a one month period giving a total dose of 2.74 × 10^9^ viable MAP. The same batches of prepared inoculum were used for all breeds.

### Ante-mortem sampling and examinations

Blood and faecal samples were collected at regular intervals (2–4 months) from each lamb. All animals were monitored by visual inspection at least three times weekly. From eight and a half months post inoculation, bodyweights visual inspections were initially carried out on a monthly basis and the frequency was increased to weekly inspections to aid identification of individuals with clinical disease.

### Necropsy and tissue collection

Sheep were culled from the experiment if they lost ≥10% of their body weight in 1 month. Any animal culled for weight loss also had a visual assessment to determine weight loss. All animals remaining at 14 months post inoculation were culled. Four inoculated animals died or were euthanised for reasons other than JD. These were Border Leicester (*n* = 2) and Merino (*n* = 2) sheep and the reasons for culling were misadventure, congenital disorder, liver disorder and one case of caseous lymphadenitis. Tissue culture data from two of these animals, both Merino, were included in the analysis, as their necropsy was conducted greater than 8 months post inoculation, a period considered to be sufficient for the infection to be detected.

Euthanasia of the animals and tissues sampled were as described by Begg et al. [15] with minor modifications. The tissues collected from each animal for culture and histology were terminal ileum, middle jejunum, posterior and middle jejunal lymph nodes and a section of the liver. Sections were either frozen at −80 °C for MAP detection or placed in 10% neutral buffered formalin.

### Histopathology

Formalin fixed tissues were embedded in paraffin, sectioned at 5 µm and stained with haematoxylin and eosin and Ziehl–Neelsen methods. Intestinal sections were graded as a score 0, 1, 2, 3a (paucibacillary) 3b (multibacillary), or 3c (severe paucibacillary) using established criteria [16]. Granulomatous lesions observed in the lymph nodes were graded as 1 (mild focal), 2 (mild multifocal) or 3 (severe multifocal to diffuse). Each animal was classified based on the highest grade of lesion observed.

### MAP detection

Culture of MAP from faeces and tissues including intestine, associated lymph nodes and liver was performed using liquid culture media M7H9C as described previously [17, 18].

### qPCR detection of MAP in faeces

Faecal samples were stored at −80 °C to ensure the integrity of the sample. Detection of MAP DNA from the samples was performed as described previously [19]. Briefly, a suspension of 1.2 g (dry) or 1.5 g (moist) faeces was prepared in 10 mL 0.85% w/v sterile saline. After vigorous shaking, this was allowed to settle for 30 min and 3–5 mL of supernatant was centrifuged at 1231 × *g* for 30 min. To the pellet, 600 μL Lysis/binding solution (597.2 μL Buffer RLT and 2.8 μL Carrier RNA; Biosprint^®^ 96 One-For-All Vet kit, Qiagen) was added, then transferred to a 2 mL screw capped tube containing 0.3 g of Zirconia/Silica beads (BioSpec Products Inc, Daintree Scientific) and disrupted using a mechanical cell disruptor/bead beater. The supernantant (400 μL) was transferred to a deep 96 well plate, with 40 μL Proteinase K and 300 μL Magnetic Bead mix (Biosprint^®^ 96 One-For-All Vet kit, Qiagen). The DNA was eluted following the “BS96 Vet 100″ instrument protocol run on an automated magnetic particle processor (BioSprint 96, Qiagen). Positive and negative faecal controls, a process control (all buffers), and an extraction plate control were included in every experiment.

MAP DNA was detected by qPCR for the IS900 gene on an Mx3000P real-time PCR instrument (Stratagene, Agilent), using SensiMix SYBR Low-ROX qPCR master mix (Bioline) with forward and reverse primers at 250 nM (MP10-1 forward 5′-ATGCGCCACGACTTGCAGCCT-3′; MP11-1 reverse 5′-GGCACGGCTCTTGTTGTAGTCG-3′). The cycling parameters were: 95 °C for 8.5 min, 40 cycles at 95 °C for 30 s, 68 °C for 60 s, and melt curve analysis from 65 to 95 °C. A standard curve of MAP genomic DNA was included in every qPCR experiment (10–0.001 pg/reaction). The criteria for positive results (≥0.001 pg MAP genomic DNA) was determined by prior validation.

### MAP specific antibody

The level of MAP specific antibodies was measured using a commercially available kit (Institut Porquier from IDEXX) following the manufacturer’s instructions. The data are presented as S/P%, which was calculated as: (OD sample − OD negative control)/(OD positive control − OD negative control) × 100.

### MAP specific IFNγ detection

The IFNγ stimulation was carried out using whole blood stimulated with MAP-specific antigen, a French pressed whole cell 316v strain of MAP(316v) or media for 48 h and the ELISA was performed as previously described [20]. On each ELISA plate sheep specific IFNγ positive and negative controls were used to calculate the SP ratio. The same batch of each control was used on all test plates. The raw data were transformed into S/P%, which was calculated as: (OD sample − OD negative control)/(OD positive control − OD negative control) × 100. The SP% of the media stimulated response was subtracted from the MAP antigen response to obtain the MAP-specific response.

### Case definitions

An animal was classified as having clinical disease if the following criteria were met: it lost ≥ 10% of its body weight over 1 month, MAP was cultured from tissues after necropsy, and histopathological lesions consistent with JD were observed.

### Statistical analysis

Contingency tables of breed with each of the binary outcome variables were created using FREQ procedure in SAS (© 2002–2012 by SAS Institute Inc., Cary, NC, USA) and cumulative incidence calculated. Log-linked binomial models were then fitted for each binary outcome variable with breed as an explanatory variable using Genmod procedure in SAS. Relative risk and its 95% confidence limits were calculated by exponentiation of the parameter estimate and their confidence limits.

Summary statistics and graphical summaries of serum antibody and IFN-γ responses were prepared to evaluate their distributions. General linear models were fitted with log transformed MAP-specific serum antibody or IFN-γ response as outcome variables and breed, months post infection and their interaction as fixed effects. Predicted means for log MAP-specific serum antibody and log IFN-γ responses for four breeds at various time points after infection were estimated and exponentiated to obtain geometric means. Standard errors of geometric means were approximated using the Delta method [21]. Assumptions of general linear models were evaluated using residual diagnostics.

All *p*-values reported in the manuscript are two sided. A 5% level of significance was considered for all analyses. Analyses were conducted using SAS Statistical program unless indicated otherwise.

## Results

### Clinical Johne’s disease

**Figure 1:**
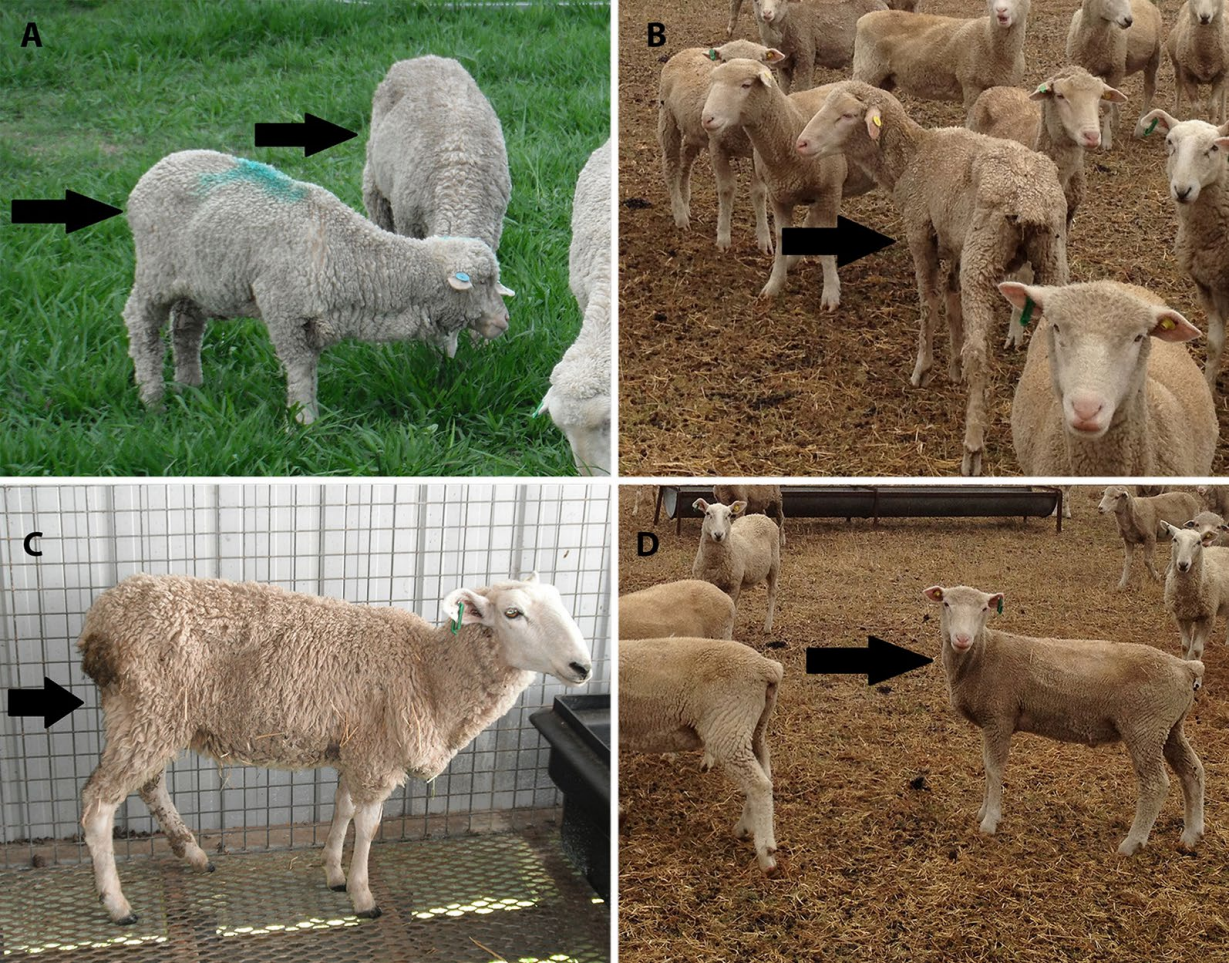
**Clinical cases of JD in different sheep breeds.** Arrows point to the clinical cases. **A** Merino, **B** Suffolk first cross Merino, **C** Border Leicester and **D** Poll Dorset; the clinical case determined by weight loss is difficult to observe by visual assessment alone in the Poll Dorset breed.

Clinical cases of JD were identified in all breeds (Figure 1). It was not possible to identify individuals developing clinical disease by visual assessment of the Poll Dorset breed, although several met the definition of a clinical case (≥ 10% loss of body weight in a month, confirmed by histopathology and culture) (Figure 1D). Clinical disease was seen more often in the Merino (42%) and Suffolk first cross Merino (36%) than in the Border Leicester (12%) and Poll Dorset (11%) sheep in the time frame examined, up to 14 months post inoculation, when the trial was terminated (Table 1). There was a significant difference in the frequency of development of clinical disease between the breeds (Table 1). The MAP inoculated Merino sheep were used as the positive control as the experimental infection model was previously validated in this breed [15]. In comparison to Merino, the Border Leicester and Poll Dorset breeds had significantly less risk of animals developing clinical disease (*p* = 0.01), but no significant difference was observed between the Merino and White Suffolk × Merino breeds (Table 1). The peak time of clinical case detection for the Merino and Suffolk first cross Merino sheep was at approximately 12 months post inoculation. The Border Leicester and Poll Dorset breeds had an increasing number of clinical cases in the final weeks of the trial, i.e. approaching 14 months post inoculation (data not shown).

**Table 1:** **Occurrence of clinical disease in four breeds of sheep 14** **months after MAP inoculation based on a trial conducted in Australia in 2012**

Breed	Total number of animals	Number of sheep developing clinical disease	Occurrence (%)	Relative risk	(95% CI)	*p* value
Merino	36	15	42	1.00^a^		
White Suffolk × Merino	36	13	36	0.87	(0.48–1.55)	0.63
Border Leicester	34	4	12	0.28	(0.10–0.77)	0.01
Poll Dorset	36	4	11	0.27	(0.10–0.73)	0.01

^a^Reference group.

### Rates of infection and dissemination

At least 45% of sheep from each breed were infected at the time of necropsy; the Merino (69%) and Suffolk first cross Merino (75%) breeds had a greater proportion of animals with viable MAP in their tissues (i.e. were infected) (Table 2), compared to Border Leicester (53%) and Poll Dorset (47%) sheep.

**Table 2:** **Occurrence of infection in four breeds of sheep at necropsy (up to 14** **months after MAP inoculation) as determined by culture from gut associated tissues**

Breed	Total number of animals	Number of sheep with MAP infection	Occurrence (%)	Relative risk	(95% CI)	*p* value
Merino	36	25	69	1.00^a^		
White Suffolk × Merino	36	27	75	1.08	(0.81–1.44)	0.60
Border Leicester	34	18	53	0.76	(0.52–1.12)	0.17
Poll Dorset	36	17	47	0.68	(0.45–1.02)	0.06

^a^Reference group.

Dissemination of MAP to tissues outside of the gut was examined by culture of a section of liver. Dissemination rates varied from 9 to 39% among the different breeds (Table 3). Both the Poll Dorset and Border Leicester breeds had significantly fewer sheep with MAP cultured from the liver *(p* = 0.03 and *p* = 0.01) compared to the Merinos (Table 3). In most cases these were the animals that developed clinical disease, although not all of the clinical cases had disseminated infection and one of the Poll Dorset animals that had disseminated infection did not have clinical disease.

**Table 3:** **Analysis of disseminated infection after MAP inoculation as determined by culture of the liver**

Breed	Total^a^	Number of sheep with disseminated MAP infection	Occurrence (%)	Relative risk	(95% CI)	*p* value
Merino	33	13	39	1.00^b^		
White Suffolk × Merino	34	13	38	0.97	(0.53–1.77)	0.92
Border Leicester	33	3	9	0.23	(0.07–0.74)	0.01
Poll Dorset	36	5	14	0.35	(0.14–0.88)	0.03

^a^Total number of animals which had liver samples cultured.

^b^Reference breed.

**Figure 2:**
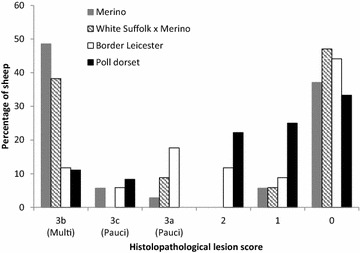
**The percentage of sheep of each breed with different histopathological lesion scores, based on the most severe lesion observed in an animal.** Animals were necropsied at 14 months post inoculation or earlier if ≥ 10% weight loss occurred over 1 month. Lesions scores: 3b or multibacillary; 3c or severe paucibacillary; 3a or paucibacillary.

Slightly fewer animals had histopathological lesions consistent with JD in their intestines than had MAP detected in their tissues. There was no significant difference in the occurrence of histopathological lesions greater than grade 1 [16] between breeds (Table 4). The Merino and Suffolk first cross Merino breeds were more likely to have multibacillary lesions or to have no lesions (lesion score 0) (Figure 2). All the breeds had a similar proportion of animals that did not develop any histopathological lesions (33–47%). The Border Leicester breed was most likely to have paucibacillary lesions but had a range of lesions from multibacillary to the least severe grade 1 lesions. The Poll Dorset breed tended to have lower grades of lesion (lesion score 0, 1 and 2).

**Table 4:** **Analysis of histopathological lesions after MAP inoculation**

Breed	Total^a^	Number of sheep with MAP histological lesions^b^	Occurrence (%)	Relative risk	(95% CI)	*p* value
Merino	35	20	57	1.00^c^		
White Suffolk × Merino	34	16	47	0.82	(0.52–1.30)	0.41
Border Leicester	34	16	47	0.82	(0.52–1.30)	0.41
Poll Dorset	36	15	42	0.73	(0.45–1.18)	0.20

^a^Total number of animals that had sections examined for histology.

^b^Animals were considered to have MAP associated histopathology if the lesion score [16] was greater than 1.

^c^Reference group.

Of the two matching intestinal and lymph node sections used for histopathological analysis (terminal ileum/posterior jejunal lymph node and middle jejunum/middle jejunal lymph node), there was a significant difference (*p* = 0.04) in the location of the most severe histological lesion between the breeds (Table 5). Data from animals with histological lesions greater than 1 were included for analysis. The Merino, Suffolk first cross Merino and Border Leicester breeds were more likely to have the most severe lesions observed in the terminal ileum or posterior jejunal lymph node. The Poll Dorset breed animals were significantly (*p* = 0.04) more likely to have the most severe lesion in the middle jejunum and/or lymph node, with a prevalence of 67%.

**Table 5:** **Analysis of the site of the most severe intestinal histopathological lesion after MAP inoculation**

Breed	Total^a^	Number of sheep with the most severe lesion in the mid JJ or LN^c^	Occurence (%)	Relative risk	(95% CI)	*p* value
Merino	20	6	30	1.00^b^		
White Suffolk × Merino	16	4	25	0.83	(0.28–2.46)	0.74
Border Leicester	16	4	25	0.83	(0.28–2.46)	0.74
Poll Dorset	15	10	67	2.22	(1.04–4.75)	0.04

^a^Any animal with a histopathological lesion greater than 1 was used for analysis [16].

^b^Reference group.

^c^Mid JJ and LN: middle jejunum/middle jejunal lymph node sections.

### Faecal shedding of MAP

**Figure 3:**
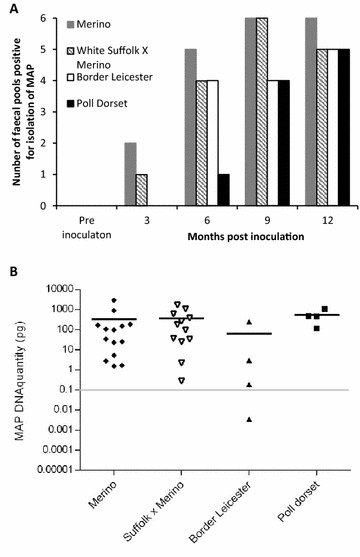
**Faecal shedding from the different breeds of sheep inoculated with MAP.** **A** The number of faecal culture positive results is shown for each breed over the course of the trial, from 6 pooled faecal cultures/breed group with 6 animals per pool. **B** Faecal shedding of MAP in sheep from each breed that developed clinical disease, as measured by qPCR. MAP DNA quantity in picograms (pg) is shown on the γ-axis on a logarithmic scale. The grey line at 0.1 pg indicates results above which are considered to be in the high range of qPCR results, approximately equivalent to > 10,000 MAP/g of faeces.

Faecal samples were pooled from 6 animals of the same breed, creating 6 pools per breed. The animals were always allocated into the same pool at all sampling points. The Merino and Suffolk first cross Merino were the only breeds to shed MAP at 3 months post inoculation (Figure 3A). As the trial progressed, the Border Leicester and Poll Dorset breeds had increasing numbers of pools with MAP detected, indicating increasing faecal shedding. At 12 months post inoculation, the number of pooled faecal cultures of the Suffolk × Merino breed decreased from 6 to 5; this was primarily due to removal of some sheep of this breed due to clinical disease.

MAP shedding by the clinically diseased sheep was examined from faecal samples collected at necropsy or at the last sampling point from each animal. The amount of MAP shed in the faeces of sheep with clinical disease was estimated using qPCR and was not significantly different between the breeds (Figure 3B).

### Host immune response to MAP

**Figure 4:**
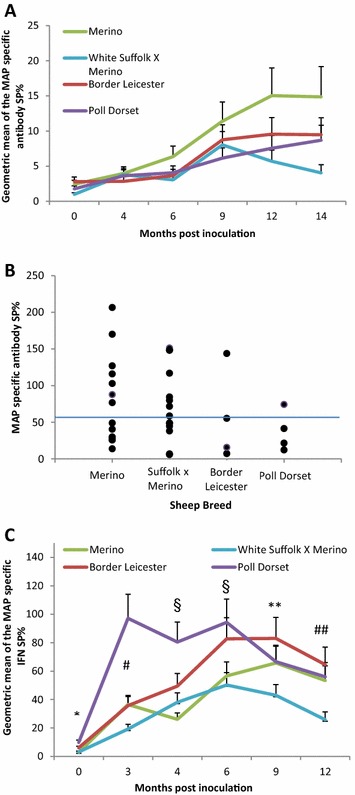
**Specific immune responses from the different breeds of sheep inoculated with MAP.** **A** MAP specific antibody responses. Data shown are Geometric means of the MAP specific SP% from the assay for each breed over the trial. Error bars indicate the standard error. ×, Significant difference between the White Suffolk × Merino animals and the Border Leicester and Merino breeds (*p* < 0.05). *, significant differences between the White Suffolk × Merino and Merino breeds (*p* < 0.05). **, significant differences between the White Suffolk × Merino and the other breeds (*p* < 0.05). **B** MAP specific antibody responses from animals with clinical disease, quantified at the last sampling taken before or at necropsy. MAP specific antibody responses were measured by a commercial ELISA (Institut Porquier from IDEXX); the blue horizontal line at SP% 55 represents the positive–negative cut point for the assay. **C** MAP specific interferon gamma responses from the different breeds of experimentally inoculated sheep. Data shown are geometric means of the MAP specific SP% from the assay. Error bars indicate the standard error. Poll Dorset and Border Leicester animals had a significantly (*p* > 0.01) greater than the Merino and White Suffolk × Merino. ^#^Poll Dorset animals had a significantly greater mean response to the other breeds (*p* > 0.0001), the White Suffolk had a significantly lower response that the Merino and Border Leicester (*p* > 0.05). ^§^Poll Dorset animal’s response significantly greater response than the Merino and White Suffolk × Merino breeds (*p* > 0.005). **The Border Leicester breed had a significantly greater response than White Suffolk × Merino (*p* > 0.01). ^##^At 12 months post inoculation the White Suffolk × Merino breed had a significantly lower response than the other breeds *(p* > 0.05).

MAP-specific serum antibody responses were measured throughout the experiment. There were significant time and breed interactions observed (*p* < 0.0001). At the sampling prior to inoculation, the Suffolk first cross Merino animals had a lower mean MAP-specific antibody response compared to the Merino and Border Leicester breeds (*p* < 0.05) (Figure 4A). The MAP specific antibody responses from the un-inoculated sheep remained at baseline levels with average SP% less than 5 for all breeds (data not shown). At 12 and 14 months post inoculation, mean responses from the Suffolk first cross Merino animals were significantly lower compared to the other breeds (*p* < 0.05) (Figure 4A). By 14 months post inoculation both the Suffolk first cross Merino and Merino breeds had had animals culled due to clinical JD, and it would be expected that the mean antibody level would wane as these sheep were removed from the study.

Examination of the antibody responses of the clinical cases from the last sampling timepoint, taken before necropsy or at the time of necropsy, indicated a wide range of responses between individual animals (Figure 4B). Half of the total combined clinically affected animals from all breeds were classified as test positive, with the remainder falling below the threshold for a positive antibody response.

The IFN-γ response showed significant breed and time interactions (*p* < 0.0001). Overall, the White Suffolk first cross Merino sheep had the lowest mean MAP specific IFN-γ response (Figure 4C). The Poll Dorset sheep had strong early responses, which were significantly different from all the other breeds at 3 months post inoculation (*p* < 0.0001) (Figure 4C). The responses from all breeds had decreased at 12 months post inoculation, with the White Suffolk first cross Merino animals having a significantly lower response (*p* < 0.05) compared to the other breeds.

## Discussion

The outcomes of this trial indicated that all of the breeds examined were susceptible to development of JD in this experimental model, and that the Merino and Suffolk first cross Merino breeds developed the disease earlier than did the other breeds. When the trial was terminated at 14 months post inoculation, 47–75% of sheep from all breeds were infected with MAP and animals of every breed had developed clinical signs and were infectious. High quantities of MAP DNA were detected in the faeces of clinical cases independent of breed. As the experimental infection model is repeatable in Merino sheep, and representative of natural infection in terms of prevalence and spectrum of final disease states [15, 22], it is likely that the results for other breeds have external validity and would apply in natural infections of similar S strains of MAP.

In this experiment the sheep were assessed until 14 months post MAP exposure and by then 25% had developed clinical disease; had the trial continued it is possible that more sheep would have developed clinical disease. In a prior trial of 2.5 years duration in (*n* = 20) Merino sheep, 7 of 8 affected sheep succumbed to clinical disease during a 4 month period commencing 14 months post inoculation, and the total proportion of clinical cases was 35% [23]. Consistent with these findings, the Merino and Suffolk first cross Merino breeds had 42 and 36% clinical cases, respectively, with disease manifesting over a period of approximately 4 months commencing 10–14 months post inoculation, while the other breeds had a lower incidence of clinical cases. It is possible that more sheep from the Poll Dorset and Border Leicester breeds may have progressed to a more severe stage or to clinical disease if the trial had continued beyond 14 months. This view is supported by the increasing number of clinical cases in the final weeks of the trial for these two breeds, the increasing number of positive faecal pools detected by faecal culture as the trial progressed, and the fact that similar numbers of sheep from these two breeds had histological lesions consistent with JD at the end of the trial compared to the Merino breed, but the lesion grades were less severe indicative of an earlier stage of disease pathogenesis.

Detection of MAP from the tissues of the different breeds of sheep indicated that there were no significant differences in infection rates between the Merino and other breeds examined. Similarly, all of the breeds had comparable numbers of animals positive for JD histopathological lesions. As has been found in previous studies, disseminated infection is normally limited to sheep with clinical disease and/or severe histopathological lesion grades [24]. In this experiment there was only one exception: a Poll Dorset animal that had no gross lesions, minor histopathological lesions in the gut and mesenteric lymph nodes but which had viable MAP in its liver.

It is accepted that the predilection site for MAP infections in ruminants is the ileum [25] and that gross and histopathological lesions are most prominent in the terminal ileum, but may extend from the caecum to duodenum [26]. This study is the first to show that the breed of animal may have a significant impact on the site where the most severe lesions were observed; in the Poll Dorset sheep histopathological lesions were more likely to be observed in the middle jejunum and/or lymph node rather than terminal ileum and posterior jejunal lymph node. Therefore, detection of JD in different breeds of sheep by histopathological examination may be improved by examining multiple sites along the ileum and jejunum.

As MAP infection in ruminants progresses, the level of faecal shedding of MAP also increases. One of the questions we aimed to answer was: do different breeds infected with the same strain and amount of MAP at the same age become equally infectious? All of the clinically affected animals, irrespective of breed, were highly infectious, although the faecal shedding of MAP in the Poll Dorset and Border Leicester breeds was slower to develop than in Merino sheep. If left unmanaged on farm, the number of clinically affected sheep will increase. For some breeds the mortalities may take longer to become apparent, possibly creating a trading risk for farmers if not diagnosed.

The early cell-mediated immune response patterns amongst the breeds support previous results demonstrating that an early low IFNγ response is associated with susceptibility to disease and faecal shedding in Merinos [27]. The breed that had the lowest number of clinically diseased and infected animals, the Poll Dorset, also had sheep with the strongest early IFNγ responses. The responses in the other breeds also support our hypothesis that the magnitude of the early IFNγ response is associated with protection. Other studies by our group have also indicated that this early cell-mediated immune response is important in the divergence of disease outcomes [28, 29].

The MAP specific serum antibody level was significantly different between breeds at later time points (> 6 months post inoculation). Breed differences in anti-MAP antibody production in cattle have been previously reported [30]. In that study, 1–2 blood samples per animal were examined in naturally infected Brahman, Angus or cross bred cows. A pure bred Brahman cow was more likely than the others to have a high antibody level or ELISA score.

Unlike the IFNγ response, the magnitude of the antibody response did not match the severity of disease outcome. The Merino and Suffolk first cross Merino sheep had similar disease outcomes but the latter had significantly lower serum anti-MAP antibody levels as the disease progressed. Interestingly, the majority of clinically diseased Border Leicester and Poll Dorset sheep would not have been diagnosed by serum ELISA tests using current recommendations for the positive–negative cut-point. This was exacerbated by the inability to detect some Poll Dorset animals with weight loss using a visual inspection. These findings have fundamental implications for disease diagnosis. Breed-specific cut points for serum antibody ELISA may need to be developed.

The sensitivity of the ELISA for detection of clinical cases was approximately 50%. As JD progresses the amount of MAP specific antibody increases especially in those animals with multibacillary lesions [31, 32], with sensitivities of ELISAs for affected sheep ranging from 36 to 85% [33]. Most of the clinical cases in this trial had multibacillary lesions, irrespective of the breed, indicating that the ELISA used in this study had a sensitivity at the lower end of the range.

One of the operational issues that occurred in this study was that the suppliers of the Border Leicester and Poll Dorset lambs provided mostly female animals. However, there are no reports of a difference in the susceptibility to develop clinical JD in relation to the sex of an animal. In humans, tuberculosis is typically observed more often in males than in females (1.9:1) although regional differences in these proportions do occur [34, 35]. In tuberculoid leprosy, the disease ratio is reversed, 0.82:1 [36]. Consequently, it is possible that there was gender bias in the results of this trial, but it is not possible to confirm this without further specific studies in sheep.

It is known that in deer of the same breed there are differences in susceptibility or resistance to MAP infection attributable to sire effects [37]. Within-breed MAP susceptibility differences are likely to occur in other ruminant species but have not been examined in detail. In this study, although the sheep of each breed were sourced from a single farm, they may have been derived from different sires. A study to examined intra and inter-breed differences would be complex and require large numbers of animals; it was beyond the scope of this trial.

In conclusion, a susceptibility to MAP infection was observed in all breeds that were examined in this study, as determined by infection and clinical disease development. However, there were differences in the disease outcomes observed: Merino and Suffolk cross Merino had more clinically affected animals in the timeframe examined; Poll Dorset and Border Leicester sheep had a slower disease progression. Importantly, all clinical cases, regardless of breed, were equally infectious, shedding large numbers of MAP. Thus for design of control programs it should be assumed that sheep of all breeds can become infectious following MAP exposure. The slower development of disease in Poll Dorset and Border Leicester sheep may provide an opportunity for farmers, as a move to these breeds may reduce environmental contamination of MAP by reduced faecal shedding, and they may have a longer economic life. On the other hand, infection could be harder to detect in these breeds due to delayed seroconversion and/or difficulty of assessing weight loss by visual means. These findings have important implications for decision making related to control and management strategies for MAP at farm and regional levels.
